# From “Gut Feeling” to Objectivity: Machine Preservation of the Liver as a Tool to Assess Organ Viability

**DOI:** 10.1007/s40472-018-0178-9

**Published:** 2018-01-20

**Authors:** Christopher J. E. Watson, Ina Jochmans

**Affiliations:** 1 Department of Surgery, University of Cambridge School of Clinical Medicine, Cambridge, UK; 2 The National Institute of Health Research (NIHR) Cambridge Biomedical Research Centre and the NIHR Blood and Transplant Research Unit (BTRU) at the University of Cambridge in collaboration with Newcastle University and in partnership with NHS Blood and Transplant (NHSBT), Cambridge, UK; 3 Laboratory of Abdominal Transplant Surgery, Department of Microbiology and Immunology, KU Leuven, Leuven, Belgium; 4 Department of Abdominal Transplant Surgery, University Hospitals Leuven, Leuven, Belgium

**Keywords:** Liver transplantation, Organ preservation, Normothermic perfusion, Viability assessment, Machine perfusion

## Abstract

**Purpose of Review:**

The purpose of this review was to summarise how machine perfusion could contribute to viability assessment of donor livers.

**Recent Findings:**

In both hypothermic and normothermic machine perfusion, perfusate transaminase measurement has allowed pretransplant assessment of hepatocellular damage. Hypothermic perfusion permits transplantation of marginal grafts but as yet has not permitted formal viability assessment. Livers undergoing normothermic perfusion have been investigated using parameters similar to those used to evaluate the liver in vivo. Lactate clearance, glucose evolution and pH regulation during normothermic perfusion seem promising measures of viability. In addition, bile chemistry might inform on cholangiocyte viability and the likelihood of post-transplant cholangiopathy.

**Summary:**

While the use of machine perfusion technology has the potential to reduce and even remove uncertainty regarding liver graft viability, analysis of large datasets, such as those derived from large multicenter trials of machine perfusion, are needed to provide sufficient information to enable viability parameters to be defined and validated .

## Introduction

The assessment of donor liver viability before transplantation has traditionally involved pre-retrieval review of the donor circumstances and biochemistry, followed by visual appraisal of the organ in situ in the donor. This has been further refined by the development of prognostic models which give an estimation of the risk of graft failure based on multivariate analysis of large donor datasets, so called donor risk indices [1•, 2–4]. These indices include factors relating to donor age, sex, race, height, cause of death (trauma, stroke, anoxia or other), bilirubin, smoking history, the location of the donor in relation to the transplant centre, whether the graft was whole, split, or a partial graft, whether from a brain dead or circulatory death donor and cold ischaemic time [1•, 3, 4].

While such indices have helped reduce uncertainty about the suitability of a liver for transplantation, they have not eliminated it. There are many reasons for this, some of which are enumerated below:

1. *Causes of death*
  While all indices have a broad category distinguishing causes of death associated with better and worse outcomes for the liver following transplantation (e.g. trauma and stroke, respectively), other causes of death may have adverse effects on graft outcomes (e.g. carbon monoxide poisoning). In addition, hepatic ischaemia prior to death (e.g. as a result of an out of hospital cardiac arrest) will have different graft outcomes depending when in the recovery from the initial ischaemic insult the organ was retrieved from the donor and transplanted.
2. *Mechanism of death*
  While donor risk indices include a variable for livers donated after circulatory death (DCD), none include any variable relating to the duration of the withdrawal phase (withdrawal of treatment to circulatory arrest) or the duration of asystole (circulatory arrest to cold in situ perfusion) which may be important outcome determinants [5]. Animal work has documented the adverse hormonal and haemodynamic changes occurring in this period which affect graft outcomes [6, 7]. Similarly, brain death in donation after brain death (DBD) donors has complex and widespread consequences that affect all organs. The autonomic reflex response to increasing intracranial pressure includes the release of massive quantities of catecholamines; this release causes peripheral vasospasm and reduced organ perfusion. In the process of brain death, hemodynamic instability worsens, with loss of sympathetic vascular tone and hypovolemia as a consequence of diabetes insipidus. Brain death also results in a range of pro-inflammatory and immune responses that affect the donor liver and impair outcome [8–10]. Variables, such as the time from brain death to donation, have been shown to affect outcomes in renal transplantation and may have similar effects on liver outcomes [11]. None of these are taken into account in the different risk indices.
3. *Pre-donation management*
  A number of different donor treatment strategies have been suggested to influence the outcomes of transplanted organs. One of the most recent is the induction of mild hypothermia (35 ± 0.5 °C) in DBD donors which has been shown to reduce delayed graft function in renal transplantation, and to be particularly beneficial to extended criteria donor kidneys [12].
4. *Steatosis*
  Steatosis is well recognised as an adverse factor, one associated with primary non-function and early allograft dysfunction [13]. It is not included in any of the above prognostic models and is difficult to quantify accurately in the donor [14].
5. *Extraction time*
  Recent work has shown that the period of time taken to extract kidneys from the donor and place them into cold storage influences early outcome significantly [15], and it is likely that the time taken to extract the liver has a similar adverse effect. Although flushed with cold preservation solution in situ, it takes some time for the liver to cool down [16], and during prolonged extraction, it is probable that some rewarming occurs that might have an effect on short- and long-term outcome.
6. *Recipient factors*

Recipient variables also affect the outcome of liver transplantation and should be kept in mind when selecting an appropriate donor liver [17, 18].

It is against this background of uncertainty that liver transplant surgeons are challenged to decide whether or not to use a liver graft, and in whom to use it, knowing also that a decision not to proceed leaves their patient at risk of death on the waiting list. In 2015 in the USA, 1673 (11%) patients died on the waiting list and 1227 (8%) were removed due to being too sick, while 703 livers were retrieved but not transplanted, representing 9.6% of all retrieved livers [19, 20]. A similar picture is seen in the UK and across the Eurotransplant region, with around 18% of patients dying or being removed from the waiting list while potentially viable DBD and DCD livers go unused [21, 22].

This review summarises some principles that might be used for viability assessment as well as recent advances and current limitations of liver graft viability assessment using extracorporeal machine perfusion technologies.

In assessing viability, it is important to independently assess the major liver cellular compartments.

## Viability Assessment of the Hepatocellular Compartment: Metabolic Zonation

**Fig. 1:**
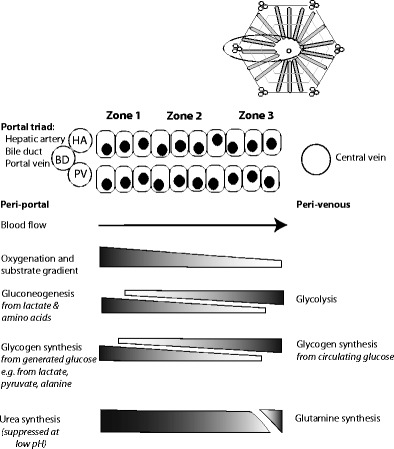
Schematic drawing illustrating metabolic zonation in the liver with reference to glucose and ammonia metabolism. Blood entering the liver lobule in vivo through hepatic artery (HA) and portal vein (PV) branches is rich in hormones, nutrients and oxygen. Periportal (zone 1) metabolic processes will include those requiring such conditions, while perivenous (zone 3) hepatocytes may preferentially include those metabolic processes that are less dependent on high levels of oxygen, for example, or those requiring products made in the periportal hepatocytes, such as urea

The range of biochemical tests examining hepatocellular function makes their interpretation complex, but we believe that the interpretation is helped by considering the tests in the context of metabolic zonation of the liver. Zonation refers to the functional specialisation of different hepatocytes along the liver lobule and is related to the exposure of zone 1 (periportal) hepatocytes to the inflow of blood with higher levels of oxygen, hormones, and metabolic substrates compared to zone 3 hepatocytes. Figure 1 illustrates the zonal distribution of some of the processes occurring in vivo. During extracorporeal perfusion, the liver is subject to different levels of oxygen and different zonal oxygen gradients, may be free of hormonal influence and is exposed to artificial concentrations of substrate depending on the perfusate used, all of which may modify the zonal behaviour.

Glucose metabolism was the first process to be identified as having zonal metabolic differences [23], with gluconeogenesis occurring in zone 1 and glycolysis in zone 3. Lactate metabolism is an oxygen and ATP-dependent process predominantly occurring in zone 1. Impaired lactate clearance implies zone 1 damage, and since zone 1 would be the last zone to be deprived of a supply of oxygenated blood, it may actually signify a pan-lobular injury. Conversely, an ischaemic injury to zones 2 and 3 may not affect lactate clearance; hence, lactate clearance is a relative insensitive marker of moderate damage, but may be a useful marker of viability. Damage to zones 2 and 3 may affect the incorporation of circulating glucose into glycogen, and therefore, perfusate glucose might be a marker of severe damage signifying impaired liver viability.

Hepatic regulation of acid-base balance depends upon the differential metabolism of glutamine along the lobule [24, 25]. Ammonia is transported as glutamine to the liver where it is hydrolysed by glutaminase to glutamate and ammonia, and the ammonia then enters the urea cycle to form urea. Glutaminase activity is largely confined to zone 1, and its activity is pH dependent, being inhibited in the presence of an acidosis. Urea synthesis can occur across zones 1, 2 and the first part of zone 3; residual ammonia is taken up by glutamine synthetase which is located in the cells immediately adjacent the central veins [26]. Interruption of ammonia metabolism will compromise the liver’s ability to regulate pH, resulting in worsening acidosis.

Other metabolic processes may be used to demonstrate liver synthetic function, such as the production of coagulation factors, albumin and complement. To be useful, there need to be rapid and sensitive assays for such metabolic products. Metabolic processes involved in drug metabolism are also distributed along the lobule, providing an opportunity to interrogate the integrity of each zone with the appropriate reagent.

Interrogating liver zones will reveal a pattern of damage, but will not necessarily give a global impression of functional liver reserve. Markers of hepatocellular damage, such as transaminase release into the perfusate, give an indication of damage, from which the amount of residual function may be inferred, but they are limited as markers of viability. Evaluating the ability of the liver to metabolise a substrate known to be metabolised by all liver zones may provide an estimate, and such assays are currently under development but are yet to be validated in an extracorporeal circuit [27]. Unlike other organs, the liver has a remarkable regenerative ability and it remains to be defined what level of injury, what threshold of residual functional capacity and which recipient circumstances are required to guarantee complete functional recovery of the liver and survival of the recipient post-transplant.

## Viability Assessment of the Cholangiocyte Compartment: Bile Biochemistry

While evaluation of hepatocellular function should enable avoidance of primary non-function of the liver, it will not predict cholangiopathy. To assess the bile duct, different physiological processes need to be monitored.

The amount of bile production has been commonly cited as a marker of liver viability [28, 29], but in our initial experience, the volume of bile produced does not appear to correlate with graft function post-transplant [30•, 31]. Bile production is a combination of two processes, bile salt-dependent secretion by biliary canaliculi (also known as the bile acid dependent canalicular fraction) and bile salt independent secretion [32]. Once in the bile ducts, the bile is subject to modification by cholangiocytes by resorptive and secretory processes, which add bicarbonate and water, and reabsorb glucose, amino acids and bile salts. The bile salt-dependent fraction of bile forms around a third of normal bile, and bile salts are either synthesised by the liver or derived from sinusoidal blood as part of the enterohepatic circulation. In an isolated perfused liver, with no supplementary bile salts added, this fraction is likely to be very small unless bile salts are added to the perfusate [33]. The bile salt-independent production of bile may also be adversely affected by ex situ perfusion. For example, hyperglycaemia, independent of diabetes, has been shown to reduce the production of bile by affecting the cholangiocyte’s absorptive processes; as the cells absorb the higher quantities of glucose, they take up more water from the bile (see below) [34]. Hence, in the presence of the high glucose concentrations seen in many liver perfusions, the volume of bile salt-independent bile produced could be reduced.

## Assessment of the Vascular Compartment: Vascular Resistance

Disruption of the endothelial cell lining and the no reflow phenomenon cause an increase in vascular resistance that reflects endothelial damage and viability, although oedema may also influence blood flow to an organ [35]. When a liver is removed from cold storage and reperfused on a machine, vascular resistance is high but falls quickly, both during hypothermic perfusion and normothermic perfusion (personal observations) [36, 37]. This resistance pattern is similar to that seen in hypothermic perfusion of kidneys, and in that setting, resistance is commonly cited as discriminating good from bad kidneys, although the evidence for a threshold value is absent [38, 39]. In a similar manner, some authors have quoted arterial and portal flows as markers of viability [40••, 41], although absolute values of flow are unhelpful without knowledge of perfusion pressure. While there are some preliminary animal data suggesting that portal (and not hepatic arterial) resistance may be discriminatory [30•], this remains to be substantiated. Portal resistance is also affected by the pressure in the hepatic veins, which varies according to which perfusion machine is used and the method of caval drainage (negative pressure or passive drainage), so absolute values will depend on the circumstance of perfusion.

## Assessment of the Immune Cell Compartment

The final compartment that contributes to reperfusion injury, and hence viability, is the immune compartment. The liver is host to numerous different leucocyte populations such as Kupffer cells and dendritic cells, all of which may respond to ischaemia-reperfusion injury by production of inflammatory proteins such as cytokines and damage-associated molecular patterns (DAMPs) [42–44]. Our unpublished observations show release of large quantities of chemokines, including interleukins-6, -8, -10, -18 and monocyte chemotactic protein 1 (MCP1/CCL2), into the perfusate reflecting activation of the immune cell compartment [42, 43]. This is in contrast to results from hypothermic oxygenated liver perfusion [45, 46]. The ability to measure the degree of immune cell activation during normothermic perfusion in real time may give another dimension to predicting viability post-transplant. It is also one area that is potentially modifiable during perfusion, with the addition of leucocyte filters to reduce the circulating numbers of emergent liver immune cells and direct chemokine inhibitors to moderate immune activation.

## Hypothermic Perfusion

One certainty is that the longer the liver remains in static cold storage, the poorer the function. It is possible that some of the effects of hypothermic storage may be mitigated by oxygenated machine perfusion. Two groups have led the evaluation of hypothermic extracorporeal liver perfusion. Guarrera and colleagues were the first to evaluate clinical hypothermic machine perfusion, and although not incorporating an oxygenator into their circuit, analysis of perfusate showed that some oxygenation was achieved passively in the organ chamber [46, 47]. The other group to pioneer hypothermic perfusion is that of Dutkowski and colleagues, who perfused the portal vein alone with actively oxygenated perfusate in a technique they termed hypothermic oxygenated perfusion (HOPE) [48]. This has been followed by Porte and colleagues performing hypothermic oxygenated perfusion of both artery and portal vein or Dual-HOPE (D-HOPE) [49]. Hypothermic extracorporeal liver perfusion has been shown to be associated with good outcomes for livers that would ordinarily be considered marginal, either because of their DCD status or because of other poor prognostic factors [46, 48, 49]. There appears to be a direct benefit in reducing expression of proinflammatory cytokines [46], down-regulation of Kupffer cell activity [45], replenishing ATP stores [50] as well as reducing vascular resistance [51, 52]. All of these may contribute to the function and marked absence of biliary complications, including cholangiopathy, in DCD livers that have experienced prolonged warm ischaemia [53•]. Replenishing oxygen at low temperature may also have the advantage of avoiding the generation of reactive oxygen species (ROS) [54, 55•], while in contrast, the generation of ROS during normothermia has been cited as a complication of aggressive oxygenation during normothermic perfusion with resultant post reperfusion syndrome and vasoplegia [31]. Whether hypothermic extracorporeal liver perfusion improves long-term outcomes is the subject of ongoing randomised controlled clinical trials (NCT01317342; NCT03124641; NCT03031067; NCT02584283).

Assessment of the liver’s functional capacity during cold perfusion is difficult, since metabolic processes are differentially affected by hypothermia and may be unrepresentative of function at normothermia. Nevertheless, biochemical analysis of the perfusate may provide insight into the degree of hepatocellular damage sustained before and during preservation [56], but simple analysis of the effluent from flushing out the cold storage solution during bench work might be equally insightful. Pacheco et al. have previously shown a relationship between the transaminase content of the effluent washed out of the graft immediately before reperfusion and the post-transplant levels in the recipient [57], and more recently, Hoyer et al. showed that the ALT levels during controlled rewarming also mirrored levels post-reperfusion in the recipient [58]. We have also demonstrated that effluent cold storage solution washed out immediately prior to normothermic perfusion correlates with ALT levels during normothermic perfusion and that perfusion levels in turn correlate with post-transplant transaminase levels [36].

## Normothermic Perfusion

The technique of normothermic extracorporeal liver perfusion (NELP) typically involves perfusing a red cell-based perfusate through hepatic artery and portal vein at physiological pressures and incorporates a heater/oxygenator into the circuit to maintain temperature and oxygenation. In contrast to hypothermic perfusion, NELP provides an opportunity to assess viability using similar biochemical assessments that are employed clinically. The viability assessment itself needs to include evaluation of both hepatocellular and biliary components of injury and function. Any functionality assessment must also recognise that the ability to function in an isolated artificial circulation of around 2 l is not the same as being able to function in vivo*.*

The absence of validated criteria for viability assessment has not prevented clinical evaluation of NELP, initially in case reports used to assess marginal grafts [59], and latterly in small clinical studies [31, 40••, 41, 60, 61]. Table 1 compares recent studies of NELP with respect to measures used to assess livers during normothermic perfusion, with reference to hepatocellular and biliary compartments. As can be seen, lactate clearance and bile output are the most common functional measures, with transaminase levels as markers of hepatocellular damage. What is more surprising is the absence of assessment of cholangiocyte integrity in many reports.

**Table 1:** Reported parameters used for the assessment of livers undergoing normothermic perfusion

Reference	Model	Hepatocellular function	Cholangiocyte function	Notes
Op den Dries [62]	Discarded human livers (*n* = 4)	*Function*: Bile output; perfusate lactate, glucose, urea, bilirubin, bicarbonate *Damage*: Perfusate ALT, GGT, Potassium	Bile bilirubin and bicarbonate concentrations Bile enzyme concentrations: GGT and LDH	Non-transplant model
Sutton [29]	Discarded human livers (*n* = 12) Extension of Op den Dries study	*Function* (assessed at 6 h): Bile output > 20 g; perfusate lactate, (glucose), albumin; sO_2_. Hepatic ATP content. Requirement for bicarbonate replacement. *Damage*: Perfusate ALT, ALP, GGT, LDH, Potassium	Bile bilirubin concentration Biliary bicarbonate concentration and pH (neither significant in study)	Recommended 2.5 h perfusion to fully assess.
Liu et al. [63]	Pigs (*n* = 10)	*Function:* Lidocaine metabolism Bile volume Oxygen consumption, pH, glucose, lactate were not discriminatory. *Damage:* Perfusate ALT, AST, LDH HA and PV resistance after 6 h.	Bile LDH, GGT, bicarbonate concentrations	10 h perfusions Samples taken at 1 h, 4 h, then 4hourly.
Nassar et al. [64]	Pigs (*n* = 20)	*Function* Urea, bile production *Damage* AST, ALT, LDH, ALP	None	60 min warm ischaemia followed by either 10 h NELP (*n* = 15) or 10 h cold storage (*n* = 5)
Reiling et al. [65]	Discarded human livers (n = 4)	*Function:* Bile production, lactate, urea, glucose *Damage* ALT	None	
Banan et al. [66]	Pigs (*n* = 18)	*Function:* Bile production; oxygen extraction; lactate; INR *Damage* Vascular resistance; ALT, AST, LDH	*Function:* Bile pH, glucose, bicarbonate *Damage:* ALP, LDH	Assessment of controlled rewarming.
Mergental et al. [41]	Clinical study: 5 transplants from 6 perfusions.	*Function:* Lactate <2.5 mmol/L; pH > 7.3 Bile production *Damage:* Hepatic and portal flows Clinical appearance	None	Recommended assessment at 3 h. Declined one liver with rising lactate. All 5 recipients well, median follow up 7 months.
Ravikumar et al. [40••]	20 clinical transplants (First in Man study)	*Monitored parameters* Perfusate pO2, pCO2, pH, glucose. HA and PV flows. Bile production.	None	Decision to use organ left to individual clinician interpretation of monitored parameters.
Selzner et al. [60]	Clinical study; 10 transplants from 12 perfusions	*Function:* Lactate; pO2, pCO2, pH; bilirubin *Damage* AST, ALT	None	No criteria stated to determine viability, but 1 graft declined due to persistently raised lactate (level not stated).
Westerkamp et al. [50]	Discarded human livers	*Function* Bile production: Glucose, lactate Bile bilirubin *Damage* HA and PV resistance ALT, AST, LDH, GGT	*Function* Bile pH and bicarbonate *Damage* GGT, LDH	Good function if bile > 2 mL/kg/h during period 1.5 to 2.5 h after start of NELP, and > 5 mL/kg/h after 2.5 h
Bral et al. [61]	Clinical study; 10 transplants from 11 perfusions	*Function:* Bile production Lactate; pO2, pCO2, pH; bilirubin; glucose *Damage* HA and PV flow AST, ALT		
Pezzati et al. [67]	Clinical case report (83 year old DBD donor)	*Function:* Bile production, lactate; pH		Post reperfusion syndrome and vasoplegia. Alive and well at 4 months.
Watson et al. [31]	Clinical study: 12 transplants from 12 perfusions of marginal grafts	*Function:* Lactate; pH; glucose *Damage* ALT	*Function* Bile pH	Post reperfusion syndrome and vasoplegia with hyperoxia.

### Hepatocellular Compartment During Normothermic Perfusion

Lactate clearance is the most widely accepted measure of liver function during normothermic perfusion, but it depends only on the viability of zone 1 hepatocytes and not zones 2 and 3. In a small volume perfusion circuit, a relatively small number of viable hepatocytes may be sufficient to clear lactate, and hence, clearance of lactate is not necessarily a marker of viability. Conversely, an inability to clear lactate may represent damage to zone 1 hepatocytes (and thus usually a pan lobular injury) or may be due to concomitant lactate production, as may happen when an aberrant lobar artery is not perfused.

**Fig. 2:**
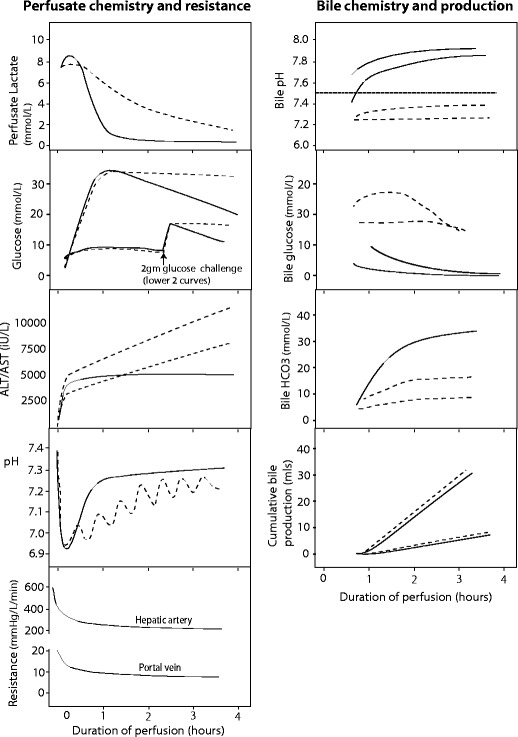
Typical normothermic perfusion profiles. The figure shows schematic graphs with typical biochemical and resistance profiles during normothermic perfusion with our interpretation given our current state of knowledge. Profiles of viable hepatocellular compartment livers are denoted by solid black lines, while dashed lines denote grafts where viability might be in doubt, due to a slow lactate clearance, persistently raised perfusate glucose, rising perfusate transaminase concentration or requirement for continued bicarbonate support to maintain pH. The graphs also show the different biochemical profiles of bile depending on the viability of the ducts, where viable cholangiocytes producing bile with a pH > 7.5, low glucose (especially relative to the high perfusate glucose) and increasing bicarbonate. To date, there is no clinical evidence in support of bile production or hepatic resistance thresholds for viability

Figure 2 shows typical biochemical profiles during NELP. The raised perfusate glucose which characterises most NELPs is likely to be in part due to glycogen breakdown (zone 3), in part due to the metabolism of lactate and in part due to glucose synthesis from circulating amino acids and pyruvate, although glucose synthesis is likely to be inhibited in the presence of high concentrations of circulating glucose. Glycogen breakdown is an oxygen-independent ATP-independent process occurring during hypothermia [68, 69]. It continues early after reperfusion during NELP, but in viable livers, the high levels of circulating glucose should block glycogenolysis and stimulate glycogenesis. As a result, perfusate glucose falls, reflecting zone 3 integrity. In some perfusions, however, the perfusate glucose may not rise, and as such may imply glycogen depletion or pan-lobular injury; in such circumstances, a glucose challenge may discriminate between the two, since in the glycogen-depleted liver, the added glucose will be metabolised or converted to glycogen. It is possible that a liver with marked disruption of zone 3 metabolism may exhibit a persistently high perfusate glucose, as the ability to incorporate glucose into glycogen is impaired. This interpretation of these observations remains to be proven.

Ammonia metabolism plays a crucial role in the liver’s ability to regulate pH. Interruption of ammonia metabolism will therefore result in worsening acidosis. Hence, the requirement for additional alkali supplementation to maintain a near physiological pH during normothermic extracorporeal liver perfusion implies a larger lobular injury affecting all zones.

### Cholangiocyte Compartment During Normothermic Perfusion

Cholangiocyte viability during NELP can be monitored by the degree to which the bile has undergone secretory and resorptive modification, and in particular, the secretion of bicarbonate to deprotonate bile acids and the removal of glucose [32, 70]. Deprotonation is believed to be necessary to prevent cholangiocyte damage by bile acids within the duct and is achieved largely by bicarbonate secretion [71, 72]. Thus, in the presence of normal cholangiocyte function, bile should have an alkali pH; our early results suggest that a pH > 7.5 is associated with viable cholangiocytes and no post-transplant cholangiopathy [36].

Glucose absorption by cholangiocytes facilitates water absorption from bile [70], and its concentration in bile should be ≤ 1 mmol/L in the context of a normal plasma glucose of 4 to 8 mmol/L [73]. Assessment of bile glucose during NELP is more complex, since the perfusate glucose concentration is frequently supra-physiological. Nevertheless, a biliary glucose ≤ 3 mmol/L or a bile/perfusate gradient ≥ 10 mmol/L was associated with viable ducts in our series [36].

The lack of viability criteria also hampers development of optimal protocols and perfusates for NELP, since without a validated viability endpoint, the results of clinical and preclinical studies are difficult to interpret. Instead, NELP has been introduced as a tool to reassure surgeons, but as such, it has not completely removed “gut feeling” in deciding whether or not to use a liver, in particular a marginal liver. In addition, there is little evidence to date that moderate periods of NELP are superior to hypothermic oxygenated machine perfusion. Indeed, the incidence of primary non-function, early allograft dysfunction and cholangiopathy in early reports of NELP contrasts with that in reports of hypothermic perfusion [31, 53•, 61, 67].

## Conclusions

Years of preclinical perfusion research, focusing on machine perfusion as a preservation method, are now translating into the clinic. While the use of machine perfusion technology has the scope to reduce and potentially even remove uncertainty regarding liver graft viability, there remains an element of gut feeling when determining whether or not to transplant a machine-perfused liver.

Although hypothermic oxygenated perfusion has been shown to permit transplantation of marginal grafts, it has not, to date, afforded the ability to verify viability before implantation. Normothermic extracorporeal liver perfusion provides more information regarding liver function, and normal biochemical parameters during perfusion are readily recognised. However, interpretation of data relating to less than ideal livers remains challenging, in particular differentiating viable from non-viable. Introduction of the technology in the absence of clear guidance regarding viability has allowed accumulation of data to inform assessment, albeit with a risk of using a graft that will not work, or which may be associated with long-term problems such as cholangiopathy. More data are needed before meaningful and accurate guidance can be produced.
